# Long-Term Clinical Implications of Atrial Fibrillation on Mortality in Patients Hospitalized with COVID-19: A Nationwide Cohort Study

**DOI:** 10.3390/jcm12206504

**Published:** 2023-10-13

**Authors:** Kyoung Ree Lim, Seunghwa Lee, Bum Sung Kim, Kwang Jin Chun

**Affiliations:** 1 Division of Infectious Disease, Department of Internal Medicine, Kyung Hee University Hospital at Gangdong, Seoul 05278, Republic of Korea; 2 Division of Cardiology, Department of Medicine, Wiltse Memorial Hospital, Suwon 16480, Republic of Korea; 3 Division of Cardiology, Department of Medicine, Konkuk University Medical Center, 120-1 Neungdong-ro, Seoul 05030, Republic of Korea; 4 Division of Cardiology, Department of Internal Medicine, Kangwon National University Hospital, Kangwon National University School of Medicine, 156, Baekryung-ro, Chuncheon 24289, Republic of Korea

**Keywords:** COVID-19, atrial fibrillation, mortality

## Abstract

Background: Atrial fibrillation (AF) increases the risk of long-term mortality in patients hospitalized with Coronavirus Disease 2019 (COVID-19), but the evidence is limited. Methods: This study used data from the Common Data Model of the Health Insurance Review and Assessment Service of Korea collected between 1 January 2020 and 30 April 2022. A total of 107,247 patients hospitalized with COVID-19 were included in this study. They were divided into two groups according to a history of AF. The primary outcome was all-cause mortality. Results: After propensity score stratification, 1919 patients with a history of AF and 105,328 patients without a history of AF who were hospitalized with COVID-19 were analyzed to determine long-term mortality. The primary outcome occurred in 99 of 1919 patients (5.2%) with a history of AF and in 1397 of 105,328 patients (1.3%) without a history of AF (hazard ratio, 1.49; 95% confidence interval 1.20–1.82; *p* < 0.01). A history of AF was also associated with an increased risk of within 30-day mortality. Conclusion: A history of AF was associated with an increased risk of long-term mortality in patients hospitalized with COVID-19. Our findings indicate the necessity for physicians to reevaluate the optimal management of patients with AF following discharge.

## 1. Introduction

Atrial fibrillation (AF) is the most common cardiac arrhythmia requiring medical therapy [1]. The incidence and prevalence of AF increase with older age [2]. AF is also more likely to develop in critically ill patients with a systemic inflammatory response or septic conditions [3,4,5]. Although Coronavirus Disease 2019 (COVID-19) is primarily a respiratory disease, the cardiovascular system is often affected [6,7]. During the COVID-19 pandemic, COVID-19-positive patients had a higher incidence of AF compared to COVID-19-negative patients [8].

Some previous studies have reported that new-onset AF during hospitalization due to COVID-19 is a risk factor for worse outcomes, including mortality [9,10]. Regarding pre-existing AF, recent studies suggested that it is associated with major cardiac adverse events or mortality [11,12,13]. However, most of these studies have been limited to single-center or small multi-center studies. Furthermore, the long-term clinical impact of a history of AF in patients hospitalized with COVID-19 has not been well evaluated. Hence, our study aimed to investigate the short-term and long-term clinical impacts of a history of AF on all-cause mortality in patients hospitalized with COVID-19 using a nationwide COVID registry.

## 2. Materials and Methods

### 2.1. Data Curation

The current data set, based on the insurance benefit claim sent to the Health Insurance Review and Assessment Service of Korea (HIRA), is comprised of all the patients who used National Health Insurance of Korea from January 2018 to April 2022. Among those people, 9,822,577 patients were selected and converted to Observational Medical Outcomes Partnership (OMOP), a common data model (CDM) by the Big Data Department of the HIRA. The name of the database is HIRA_CMD, and the used platform is Oracle. We used the database shared in the form of OMOP-CDM, which has been established as a multi-stakeholder, interdisciplinary collaborative to create open-source solutions that bring out the value of observational health data through large-scale analytics [14]. This was a retrospective observational cohort study conducted in accordance with the principles of the Declaration of Helsinki. The Institutional Review Board of Kangwon National University Hospital approved the study protocol (KNUH-2022-07-015). Informed consent was waived due to the retrospective nature of the study.

### 2.2. Cohort Definitions and Outcomes

Patients were entered into this cohort if they met the following criteria: they were 20 years of age or older, had no prior history of myocardial infarction or heart failure, and had been admitted to the hospital due to COVID-19. Hospitalized patients with COVID-19 were divided into two cohorts. The target cohort comprised patients with a history of AF, while the comparator cohort consisted of patients with no history of AF. Baseline characteristics were retrieved from the Observational Health Data Sciences and Informatics (OHDSI) CDM. The primary outcome was all-cause mortality. The secondary outcome was major adverse cardiac and cerebrovascular events (MACCE), which were defined as a composite of all-cause mortality, acute myocardial infarction, or stroke. To assess both short-term and long-term impacts, we conducted separate analyses of clinical outcomes within the 30-day period and beyond after a diagnosis of COVID-19.

### 2.3. Statistics

Analysis tools for the OMOP-CDM are built in the interactive analysis platform ATLAS and the Observational Health Data Sciences and Informatics (OHDSI) Methods Library R packages version 3.5.1 (R Foundation for Statistical Computing, Vienna, Austria). OHDSI’s open-source software (ATLAS version 2.7.6) is publicly available on the GitHub repository (https://github.com/OHDSI/). In addition, concept sets, which we used to define baseline characteristics and study outcomes, are also available (https://github.com/OHDSI/COVID-19/). The data was accessed on 20 December 2022. A Cox regression analysis was used to evaluate all-cause mortality and other clinical outcomes according to a history of AF. Kaplan–Meier estimates were used to construct survival curves and compared with the log-rank test. To retain a large sample size and maximize the study power while maintaining a balance in covariates between the two groups, we conducted rigorous adjustment for differences in baseline and lesion characteristics of patients using the weighted Cox proportional-hazards regression models with propensity score (PS) stratification [15]. All tests were two-tailed, and *p* < 0.05 was considered statistically significant.

## 3. Results

**Figure 1:**
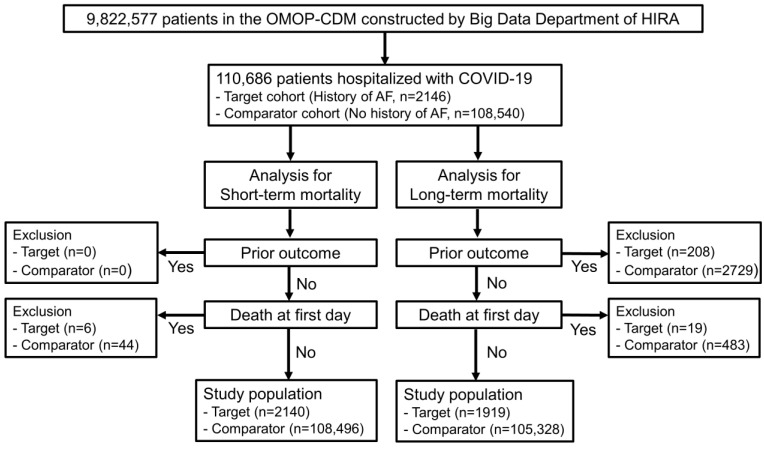
Attrition of analysis on short-term and long-term all-cause mortality. OMOP, Observational Medical Outcomes Partnership; CDM, Common Data Model; HIRA, Health Insurance Review and Assessment Service of Korea.

Among 9,822,577 patients converted to OMOP-CDM by the Big Data Department of HIRA, a total of 110,636 and 107,247 hospitalized patients with COVID-19 were analyzed for short-term and long-term mortality, respectively. Among these hospitalized COVID-19 patients, 2140 patients with a history of AF and 108,496 patients without a history of AF were analyzed for short-term mortality (Figure 1). In addition, 1919 patients with a history of AF and 105,328 patients without a history of AF were also analyzed for long-term mortality (Figure 1).

### 3.1. Baseline Clinical Characteristics

The baseline clinical characteristics of the study population analyzed for short-term and long-term mortality are shown in Table 1 and Table 2, and we found no significant imbalances in the baseline variables after propensity score stratification between the two groups (Supplementary Figures S1 and S2). The baseline characteristics of the patients analyzed for short-term and long-term MACCE, myocardial infarction, or stroke were shown in Supplementary Table S1, Table S2, Table S3, Table S4, Table S5, and Table S6, respectively.

### 3.2. Clinical Outcomes within 30 Days

**Figure 2:**
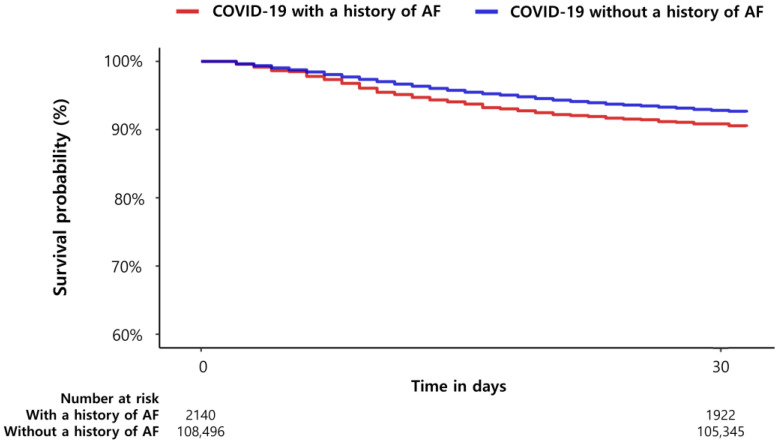
Kaplan–Meier estimates of short-term all-cause mortality according to a history of AF.

The primary outcome occurred in 205 of 2140 patients (9.6%) with a history of AF and in 2746 of 108,496 patients (2.5%) without a history of AF. After PS stratification, a history of AF was associated with an increased risk of all-cause mortality (hazard ratio [HR], 1.30; 95% confidence interval [CI], 1.13–1.50; *p* < 0.01, Table 1). Kaplan–Meier estimates of all-cause mortality according to a history of AF are presented in Figure 2. The incidence of MACCE was higher in patients with a history of AF compared with those without a history of AF (HR 1.48, 95% CI 1.20–1.80, *p* < 0.01, Table 3) after PS stratification. A history of AF was associated with higher rates of acute myocardial infarction (0.9% versus 0.3%) and stroke (1.1% versus 0.3%). The unadjusted hazard ratio for acute myocardial infarction was 2.87 (95% CI 1.69–4.52), and for stroke was 3.29 (95% CI 1.75–5.58) for patients with versus without a history of AF. However, statistical significance was fully attenuated for acute myocardial infarction (HR 1.23, 95% CI 0.72–1.95, *p* = 0.42) and for stroke (HR 1.08, 95% CI 0.57–1.84, *p* = 0.80) after adjusting for demographics and clinical comorbidities.

### 3.3. Clinical Outcomes after 30 Days

**Figure 3:**
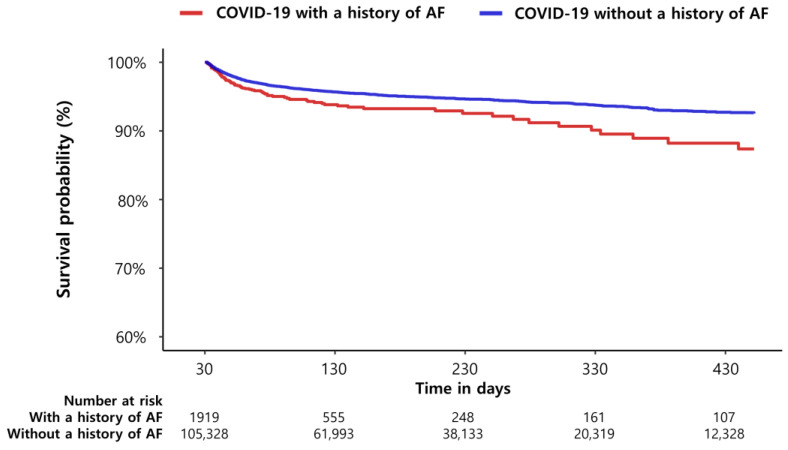
Kaplan–Meier estimates of long-term all-cause mortality according to a history of AF.

The primary outcome occurred in 99 of 1919 patients (5.2%) with a history of AF and in 1397 of 105,328 patients (1.3%) without a history of AF during the observation periods. Incidence of all-cause mortality was significantly higher in patients with a history of AF after PS stratification (HR 1.49, 95% CI 1.20–1.82, *p* < 0.01, Table 4). The Kaplan–Meier estimates of all-cause mortality according to a history of AF are shown in Figure 3. A history of AF was associated with higher rates of MACCE (4.1% versus 1.4%), acute myocardial infarction (<0.2% versus 0.1%), and stroke (0.5% versus 0.4%). The unadjusted hazard ratio for MACCE was 3.93 (95% CI 2.77–5.39), acute myocardial infarction was 4.42 (95% CI 1.35–10.58), and for stroke was 1.94 (95% CI 0.69–4.20) for patients with versus without a history of AF. However, statistical significance was fully attenuated for MACCE (HR 1.20, 95% CI 0.85–1.65, *p* = 0.28), acute myocardial infarction (HR 2.20, 95% CI 0.66–5.48, *p* = 0.14), and for stroke (HR 0.72, 95% CI 0.26–1.57, *p* = 0.48) after adjusting for demographics and clinical comorbidities.

## 4. Discussion

The main finding of this study was that a history of AF was associated with an increased risk of long-term mortality as well as short-term mortality in patients hospitalized due to COVID-19. Our findings suggest that the poor prognostic effect related to AF was not limited to short-term periods but was maintained during long-term follow-up.

It is well known that AF is an independent risk factor for worse outcomes in critically ill patients, especially those with severe sepsis or septic shock [16,17]. In particular, new-onset AF is associated with an increased risk of in-hospital mortality in critically ill patients [16,18]. In COVID-19 patients, several studies have reported that AF is an independent risk factor for short-term mortality [9,10,12,13,19,20]. However, the contribution to mortality according to AF type (new-onset AF or history of AF) is inconsistent. One study reported that there was no significant difference in mortality between patients with a history of AF and new-onset AF [20]. In contrast, other studies have shown that patients with new-onset AF had increased mortality compared to those with a history of AF [9,10,12]. Our study focused on patients with a history of AF, which was associated with an increased risk of short- and long-term mortality. Our study did not address whether AF recurred in patients with a history of AF during hospitalization, but a prior study reported that patients with a history of AF had a higher mortality rate than those without a history of AF, regardless of AF recurrence [12].

To date, the long-term impacts of AF on mortality in patients hospitalized with COVID-19 have not been well evaluated. There have only been a few studies on the long-term impacts of AF, but the follow-up durations were limited to 3 ~ 6 months [20,21]. One study reported that AF increased the long-term risk of death (180 days after hospital discharge) in patients hospitalized with COVID-19 [20]. This study also showed that there was no significant difference between pre-existing AF and new-onset AF on long-term mortality. However, the other study reported that new-onset AF on admission was associated with an increased risk of intubation and transfer to the intensive care unit but did not affect long-term mortality [21]. In our study, a history of AF in patients hospitalized due to COVID-19 was associated with an increased risk of mortality during the long-term follow-up period as well as within 30 days of a COVID-19 diagnosis. These findings suggest that patients with AF require close observation and optimal medication after hospital discharge. Furthermore, a study addressing the clinical management of new-onset AF during COVID-19 has suggested that it should not be regarded merely as an incidental occurrence in the course of COVID-19. Instead, it should be managed according to the standard practice for AF [22].

Severe acute respiratory syndrome coronavirus-2 (SARS-CoV-2) infects human cells binding to angiotensin-converting enzyme 2 (ACE2) [23]. It has been suggested that high levels of ACE2 might be susceptible to COVID-19 [23]. Plasma ACE2 activity is higher in patients with heart failure and hypertension, which is associated with atrial structural remodeling [13,23]. AF is also significantly associated with atrial structural remodeling and could be susceptible to COVID-19 [13,23].

COVID-19 causes systemic inflammation and cytokine activation, which could cause myocardial injury [24]. It was reported that levels of inflammatory and cardiac involvement markers such as procalcitonin, IL-6, NT-pro-BNP, and hs cTnI are higher in patients with AF compared to those without AF [20]. Several of these factors may be associated with worse outcomes in hospitalized patients with AF. Furthermore, the risk factors of AF were related to the risk factors of ischemic stroke [25,26]. Although our study did not show the difference in the incidence of stroke during the long-term follow-up period, these risk factors may have contributed to the poor prognosis in patients with a history of AF. However, the underlying mechanism by which AF contributes to increased long-term mortality remains unclear.

Previous studies have reported that the prevalence of a history of AF patients hospitalized due to COVID-19 ranged between 5% and 20% [27,28,29,30]. In our study, the prevalence of a history of AF was 2%, which is significantly lower than that reported in prior studies. This discrepancy could be caused by the characteristics of the enrolled patients. Previous studies were single- or multi-center studies that enrolled between 400 and 4000 patients. Since our study was a nationwide cohort study, all patients hospitalized with COVID-19 in South Korea were enrolled. Therefore, our study population was likely relatively younger and had fewer comorbid conditions than patients in previous studies.

This study had several limitations. First, this was a retrospective observational study. Although we performed propensity score stratification, potential confounders might have been present. Second, we used a CDM. Therefore, we could not analyze patient-specific data, including laboratory data, imaging studies, or electrocardiograms. We also could not identify whether AF recurred or not. Furthermore, we could not classify the patients with COVID-19 according to the severity. This study would have been strengthened if the impacts of AF on all-cause mortality had been analyzed according to the severity of COVID-19. Third, we could not evaluate whether the patients with a history of AF continued using non-vitamin K oral anticoagulants or could not use antiviral agents due to maintenance of anticoagulants during hospitalization. We also could not evaluate adherence to anticoagulation treatment after hospital discharge. Fourth, the prevalence of AF (1.9%) in this study was quite lower than the rates reported by previous studies. As mentioned above, this study was a large-scale, nationwide cohort study that included all patients hospitalized due to COVID-19 in South Korea. Therefore, the study population might be younger and have fewer comorbidities than populations of previous studies.

## 5. Conclusions

A history of AF in patients hospitalized with COVID-19 was associated with a higher risk of mortality during both short- and long-term follow-up periods. These findings suggest that AF is an important risk factor in predicting mortality, and more careful management of these patients should be required both on admission and after hospital discharge. It is imperative that physicians should reevaluate the optimal management of patients with AF following discharge.

## Figures and Tables

**Table 1:** The baseline characteristics of patients analyzed for all-cause mortality within 30 days of a COVID-19 diagnosis.

	Before PS Adjustment			After PS Adjustment		
	History of AF	No History of AF	SMD	History of AF	No History of AF	SMD
	(N = 2140)	(N = 108,496)		(N = 2140)	(N = 108,496)	
Age group						
20–24	<0.2	7.1	−0.37	0.8	6.9	−0.32
25–29	0.3	7.2	−0.37	15.1	7.1	0.26
30–34	0.4	5.9	−0.32	11.0	5.8	0.19
35–39	0.7	6.3	−0.31	1.8	6.2	−0.22
40–44	1.1	6.8	−0.30	5.6	6.7	−0.04
45–49	0.9	7.0	−0.32	7.2	6.9	0.01
50–54	1.6	8.0	−0.30	8.3	7.9	0.02
55–59	3.3	7.7	−0.19	7.7	7.6	0.00
60–64	7.3	9.3	−0.07	7.1	9.2	−0.08
65–69	9.1	7.8	0.05	5.6	7.8	−0.09
70–74	12.5	6.1	0.22	6.0	6.3	−0.01
75–79	15.3	4.9	0.35	5.5	5.1	0.02
80–84	19.8	6.1	0.42	6.8	6.4	0.02
85–89	17.5	5.5	0.38	6.0	5.7	0.01
90–94	7.9	3.2	0.21	4.2	3.3	0.05
95–99	1.8	1.0	0.07	1.1	1.0	0.01
100–104	0.3	0.2	0.03	0.2	0.2	0.00
Gender: Female	49.7	52.2	−0.05	42.3	52.3	−0.20
Medical history: General						
Acute respiratory disease	53.9	49.8	0.08	55.1	49.8	0.11
Chronic liver disease	8.2	4.0	0.17	3.8	4.1	−0.01
Chronic obstructive lung disease	10.1	2.7	0.31	3.4	2.8	0.04
Crohn’s disease	0.3	0.1	0.04	<0.1	0.1	−0.01
Dementia	45.1	17.1	0.64	19.4	17.6	0.05
Depressive disorder	33.4	16.0	0.41	16.3	16.3	0.00
Diabetes mellitus	46.8	21.8	0.55	19.8	22.2	−0.06
Gastroesophageal reflux disease	50.8	30.1	0.43	26.4	30.4	−0.09
Gastrointestinal hemorrhage	8.0	2.8	0.23	3.4	2.8	0.03
Hyperlipidemia	70.3	34.3	0.77	34.0	35.0	−0.02
Hypertensive disorder	84.3	37.1	1.10	39.7	38.0	0.03
Lesion of liver	5.7	2.9	0.14	3.8	3.0	0.05
Obesity	0.2	0.2	0.01	0.6	0.2	0.06
Osteoarthritis	24.1	14.5	0.25	11.9	14.6	−0.08
Pneumonia	27.6	11.6	0.41	11.4	11.8	−0.01
Psoriasis	0.8	0.8	0.01	0.4	0.8	−0.04
Renal impairment	21.8	4.6	0.52	7.4	4.8	0.11
Rheumatoid arthritis	2.8	1.5	0.09	1.5	1.5	0.00
Schizophrenia	2.5	3.6	−0.06	2.1	3.6	−0.09
Ulcerative colitis	0.5	0.2	0.06	0.6	0.2	0.07
Urinary tract infectious disease	19.7	7.7	0.36	12.7	7.8	0.16
Visual system disorder	41.4	31.7	0.20	44.0	31.9	0.25
Medical history: Cardiovascular disease						
Cerebrovascular disease	17.9	5.7	0.38	7.1	5.9	0.05
Peripheral vascular disease	20.2	9.7	0.30	11.3	9.9	0.05
Pulmonary embolism	7.1	1.2	0.30	2.1	1.2	0.07
Venous thrombosis	7.8	2.8	0.23	3.4	2.8	0.04
Medical history: Neoplasms						
Hematologic neoplasm	1.4	0.5	0.09	0.7	0.5	0.02
Malignant lymphoma	0.5	0.2	0.06	0.2	0.2	0.01
Malignant neoplasm of anorectum	0.8	0.3	0.07	0.2	0.3	−0.02
Malignant neoplastic disease	12.5	5.9	0.23	8.4	6.0	0.09
Malignant tumor of breast	0.3	0.6	−0.05	<0.1	0.6	−0.09
Malignant tumor of colon	1.2	0.5	0.08	0.5	0.5	0.00
Malignant tumor of lung	0.8	0.4	0.06	0.3	0.4	−0.02
Malignant tumor of urinary bladder	0.4	0.2	0.04	0.3	0.2	0.02
Primary malignant neoplasm of prostate	2.2	0.6	0.14	0.8	0.6	0.02

Data are presented as %. PS, propensity score; AF, atrial fibrillation; SMD, standardized mean difference. If the absolute value of SMD is less than 0.1 after PS adjustment, it is considered that the baseline difference is adequately balanced.

**Table 2:** The baseline characteristics of patients analyzed for all-cause mortality at more than 30 days after a COVID-19 diagnosis.

	Before PS Adjustment			After PS Adjustment		
	History of AF	No History of AF	SMD	History of AF	No History of AF	SMD
	(N = 1919)	(N = 105,328)		(N = 1919)	(N = 105,328)	
Age group						
20–24	<0.2	7.1	−0.37	2.0	7.1	−0.24
25–29	0.3	7.2	−0.37	12.8	7.3	0.18
30–34	0.4	5.9	−0.32	11.1	6.0	0.18
35–39	0.7	6.3	−0.31	4.2	6.4	−0.10
40–44	1.1	6.8	−0.30	7.8	6.9	0.04
45–49	0.9	7.0	−0.32	2.8	7.1	−0.20
50–54	1.6	8.0	−0.30	9.9	8.1	0.06
55–59	3.3	7.7	−0.19	8.4	7.8	0.02
60–64	7.3	9.3	−0.07	6.1	9.4	−0.12
65–69	9.1	7.8	0.05	6.0	7.9	−0.07
70–74	12.5	6.1	0.22	6.4	6.2	0.01
80–84	19.8	6.1	0.42	6.6	6.0	0.03
85–89	17.5	5.5	0.38	5.3	5.2	0.00
90–94	7.9	3.2	0.21	3.8	2.9	0.05
95–99	1.8	1.0	0.07	1.5	0.8	0.06
100–104	0.3	0.2	0.03	0.2	0.1	0.02
Gender: Female	49.7	52.2	−0.05	44.9	52.2	−0.14
Medical history: General						
Acute respiratory disease	53.9	49.8	0.08	61.9	49.9	0.24
Chronic liver disease	8.2	4.0	0.17	5.4	4.0	0.06
Chronic obstructive lung disease	10.1	2.7	0.31	3.4	2.5	0.05
Crohn’s disease	0.3	0.1	0.04	<0.1	0.1	0.00
Dementia	45.1	17.1	0.64	18.4	16.3	0.06
Depressive disorder	33.4	16.0	0.41	20.4	15.7	0.12
Diabetes mellitus	46.8	21.8	0.55	21.8	21.5	0.01
Gastroesophageal reflux disease	50.8	30.1	0.43	32.6	30.2	0.05
Gastrointestinal hemorrhage	8.0	2.8	0.23	6.3	2.7	0.18
Hyperlipidemia	70.3	34.3	0.77	43.1	34.5	0.18
Hypertensive disorder	84.3	37.1	1.10	43.1	36.8	0.13
Lesion of liver	5.7	2.9	0.14	4.8	2.9	0.10
Obesity	0.2	0.2	0.01	0.5	0.2	0.05
Osteoarthritis	24.1	14.5	0.25	13.4	14.5	−0.03
Pneumonia	27.6	11.6	0.41	10.6	10.9	−0.01
Psoriasis	0.8	0.8	0.01	1.5	0.8	0.07
Renal impairment	21.8	4.6	0.52	7.4	4.4	0.13
Rheumatoid arthritis	2.8	1.5	0.09	2.7	1.5	0.08
Schizophrenia	2.5	3.6	−0.06	2.0	3.6	−0.10
Ulcerative colitis	0.5	0.2	0.06	1.6	0.2	0.16
Urinary tract infectious disease	19.7	7.7	0.36	10.2	7.4	0.10
Visual system disorder	41.4	31.7	0.20	43.3	31.9	0.24
Medical history: Cardiovascular disease						
Cerebrovascular disease	17.9	5.7	0.38	7.9	5.6	0.09
Peripheral vascular disease	20.2	9.7	0.30	10.2	9.6	0.02
Pulmonary embolism	7.1	1.2	0.30	2.1	1.1	0.08
Venous thrombosis	7.8	2.8	0.23	5.5	2.8	0.14
Medical history: Neoplasms						
Hematologic neoplasm	1.4	0.5	0.09	0.5	0.5	0.00
Malignant lymphoma	0.5	0.2	0.06	0.1	0.2	−0.01
Malignant neoplasm of anorectum	0.8	0.3	0.07	0.2	0.3	−0.01
Malignant neoplastic disease	12.5	5.9	0.23	7.9	5.7	0.09
Malignant tumor of breast	0.3	0.6	−0.05	<0.1	0.7	−0.10
Malignant tumor of colon	1.2	0.5	0.08	0.4	0.5	0.00
Malignant tumor of lung	0.8	0.4	0.06	0.2	0.3	−0.02
Malignant tumor of urinary bladder	0.4	0.2	0.04	1.3	0.2	0.13
Primary malignant neoplasm of prostate	2.2	0.6	0.14	0.9	0.6	0.04

Data are presented as %. PS, propensity score; AF, atrial fibrillation; SMD, standardized mean difference. If the absolute value of SMD is less than 0.1 after PS adjustment, it is considered that the baseline difference is adequately balanced.

**Table 3:** Short-term clinical outcomes according to a history of AF.

Outcomes			Before Propensity Score Stratification		After Propensity Score Stratification	
All-cause mortality (*n*, %)	History of AF (N = 2140)	No history of AF (N = 108,496)	Unadjusted HR (95% CI)	*p* value	Adjusted HR (95% CI)	*p* value
	205 (9.6)	2746 (2.5)	3.94 (3.41–4.52)	<0.01	1.30 (1.13–1.50)	<0.01
MACCE (*n*, %)	History of AF (N = 984)	No history of AF (N = 92,113)	Unadjusted HR (95% CI)	*p* value	Adjusted HR (95% CI)	*p* value
	98 (10.0)	2030 (2.2)	4.72 (3.83–5.74)	<0.01	1.48 (1.20–1.80)	<0.01
Acute Myocardial infarction (*n*, %)	History of AF (N = 1959)	No history of AF (N = 106,387)	Unadjusted HR (95% CI)	*p* value	Adjusted HR (95% CI)	*p* value
	17 (0.9)	325 (0.3)	2.87 (1.69–4.52)	<0.01	1.23 (0.72–1.95)	0.42
Stroke (*n*, %)	History of AF (N = 1109)	No history of AF (N = 93,722)	Unadjusted HR (95% CI)	*p* value	Adjusted HR (95% CI)	*p* value
	12 (1.1)	315 (0.3)	3.29 (1.74–5.58)	<0.01	1.08 (0.57–1.84)	0.80

AF, atrial fibrillation; MACCE, major adverse cardiac and cerebrovascular events.

**Table 4:** Long-term clinical outcomes according to a history of AF.

Outcomes			Before Propensity Score Stratification		After Propensity Score Stratification	
All-cause mortality (*n*, %)	History of AF (N = 1919)	No history of AF (N = 105,328)	Unadjusted HR (95% CI)	*p* value	Adjusted HR (95% CI)	*p* value
	99 (5.2)	1397 (1.3)	5.12 (4.15–6.25)	<0.01	1.49 (1.20–1.82)	<0.01
MACCE (*n*, %)	History of AF (N = 877)	No history of AF (N = 89,807)	Unadjusted HR (95% CI)	*p* value	Adjusted HR (95% CI)	*p* value
	36 (4.1)	1263 (1.4)	3.93 (2.77–5.39)	<0.01	1.20 (0.85–1.65)	0.28
Acute Myocardial infarction (*n*, %)	History of AF (N = 1747)	No history of AF (N = 103,126)	Unadjusted HR (95% CI)	*p* value	Adjusted HR (95% CI)	*p* value
	<5 (<0.2)	94 (0.1)	4.42 (1.35–10.58)	<0.01	2.20 (0.66–5.48)	0.14
Stroke (*n*, %)	History of AF (N = 995)	No history of AF (N = 91,501)	Unadjusted HR (95% CI)	*p* value	Adjusted HR (95% CI)	*p* value
	5 (0.5)	381 (0.4)	1.94 (0.69–4.20)	0.15	0.72 (0.26–1.57)	0.48

AF, atrial fibrillation; MACCE, major adverse cardiac and cerebrovascular events.

## Data Availability

HIRA reserves the right to share data.

## Supplementary Materials

The following supporting information can be downloaded at https://www.mdpi.com/article/10.3390/jcm12206504/s1, Figure S1. (A) Propensity score, (B) covariate balance of the analysis of short-term mortality. Figure S2. (A) Propensity score, (B) covariate balance of the analysis of long-term mortality. Table S1. The baseline characteristics of patients analyzed for MACCE within 30 days of a COVID-19 diagnosis. Table S2. The baseline characteristics of patients analyzed for MACCE at more than 30 days after a COVID-19 diagnosis. Table S3. The baseline characteristics of patients analyzed for acute myocardial infarction within 30 days of a COVID-19 diagnosis. Table S4. The baseline characteristics of patients analyzed for acute myocardial infarction at more than 30 days after a COVID-19 diagnosis. Table S5. The baseline characteristics of patients analyzed for stroke within 30 days of a COVID-19 diagnosis. Table S6. The baseline characteristics of patients analyzed for stroke at more than 30 days after a COVID-19 diagnosis.
