# Supplementary material for: Long-Term Clinical Implications of Atrial Fibrillation on Mortality in Patients Hospitalized with COVID-19: A Nationwide Cohort Study
Source: J Clin Med. 2023 Oct 13;12(20):6504. doi: 10.3390/jcm12206504 (PMC10607130; doi:10.3390/jcm12206504)
Supplement: Supplementary file 1 [file jcm-12-06504-s001.zip › Supplementary_Tables S1-S6.pdf]

Supplementary Tables

Table S1. The baseline characteristics of patients analyzed for MACCE within 30 days of a COVID-19 diagnosis.

	**Before PS adjustment**			**After PS adjustment**		
	**History of AF (N=984)**	**No history of AF (N=92,113)**	**SMD**	**History of AF (N=984)**	**No history of AF (N=92,113)**	**SMD**
Age group						
20 - 24	<0.2	7.1	-0.37	1.0	8.2	-0.35
25 - 29	0.3	7.2	-0.37	10.2	8.3	0.06
30 - 34	0.4	5.9	-0.32	21.3	6.9	0.42
35 - 39	0.7	6.3	-0.31	3.7	7.2	-0.15
40 - 44	1.1	6.8	-0.30	5.2	7.8	-0.11
45 - 49	0.9	7.0	-0.32	5.5	7.9	-0.09
50 - 54	1.6	8.0	-0.30	8.7	8.7	0.00
55 - 59	3.3	7.7	-0.19	9.9	8.1	0.06
60 - 64	7.3	9.3	-0.07	8.1	9.2	-0.04
65 - 69	9.1	7.8	0.05	4.4	7.3	-0.12
70 - 74	12.5	6.1	0.22	4.8	5.3	-0.02
75 - 79	15.3	4.9	0.35	3.6	3.6	0.00
80 - 84	19.8	6.1	0.42	5.3	4.2	0.05
85 - 89	17.5	5.5	0.38	4.4	3.8	0.03
90 - 94	7.9	3.2	0.21	3.2	2.5	0.04
95 - 99	1.8	1.0	0.07	0.7	0.9	-0.02
100 - 104	0.3	0.2	0.03	<0.2	0.2	0.01
Gender: Female	49.7	52.2	-0.05	51.7	52.2	-0.01
Medical history: General						

Acute respiratory disease	53.9	49.8	0.08	61.9	51.0	0.22
Chronic liver disease	8.2	4.0	0.17	4.2	3.7	0.02
Chronic obstructive lung disease	10.1	2.7	0.31	2.7	2.0	0.05
Crohn's disease	0.3	0.1	0.04	<0.2	0.1	-0.03
Dementia	45.1	17.1	0.64	12.6	11.5	0.03
Depressive disorder	33.4	16.0	0.41	13.8	12.8	0.03
Diabetes mellitus	46.8	21.8	0.55	17.3	18.6	-0.03
Gastroesophageal reflux disease	50.8	30.1	0.43	25.2	28.9	-0.08
Gastrointestinal hemorrhage	8.0	2.8	0.23	3.5	2.3	0.07
Hyperlipidemia	70.3	34.3	0.77	31.7	30.2	0.03
Hypertensive disorder	84.3	37.1	1.10	32.0	30.5	0.03
Lesion of liver	5.7	2.9	0.14	4.1	2.7	0.07
Obesity	0.2	0.2	0.01	0.3	0.2	0.02
Osteoarthritis	24.1	14.5	0.25	10.9	13.8	-0.09
Pneumonia	27.6	11.6	0.41	8.7	9.8	-0.04
Psoriasis	0.8	0.8	0.01	0.7	0.7	-0.01
Renal impairment	21.8	4.6	0.52	6.6	3.3	0.16
Rheumatoid arthritis	2.8	1.5	0.09	1.6	1.4	0.01
Schizophrenia	2.5	3.6	-0.06	1.8	3.6	-0.11
Ulcerative colitis	0.5	0.2	0.06	0.8	0.1	0.09
Urinary tract infectious disease	19.7	7.7	0.36	6.1	6.1	0.00
Visual system disorder	41.4	31.7	0.20	51.9	31.3	0.42
Medical history: Cardiovascular disease						
Cerebrovascular disease	17.9	5.7	0.38	2.0	2.4	-0.03
Peripheral vascular disease	20.2	9.7	0.30	7.3	8.1	-0.03
Pulmonary embolism	7.1	1.2	0.30	1.9	1.0	0.07

Venous thrombosis	7.8	2.8	0.23	3.9	2.5	0.08
Medical history: Neoplasms						
Malignant lymphoma	0.5	0.2	0.06	<0.2	0.2	-0.01
Malignant neoplasm of anorectum	0.8	0.3	0.07	0.3	0.3	0.00
Malignant neoplastic disease	12.5	5.9	0.23	9.3	5.5	0.14
Malignant tumor of breast	0.3	0.6	-0.05	<0.2	0.7	-0.10
Malignant tumor of colon	1.2	0.5	0.08	0.4	0.4	-0.01
Malignant tumor of lung	0.8	0.4	0.06	0.4	0.3	0.01
Malignant tumor of urinary bladder	0.4	0.2	0.04	0.7	0.2	0.07
Primary malignant neoplasm of prostate	2.2	0.6	0.14	0.5	0.5	0.01

Data are presented as %.

PS, propensity score; AF, atrial fibrillation; SMD, standardized mean difference

If the absolute value of SMD is less than 0.1 after PS adjustment, it is considered that baseline difference is adequately balanced

Table S2. The baseline characteristics of patients analyzed for MACCE at more than 30 days after a COVID-19 diagnosis.

	**Before PS adjustment**			**After PS adjustment**		
	**History of AF (N=877)**	**No history of AF (N=89,807)**	**SMD**	**History of AF (N=877)**	**No history of AF (N=89,807)**	**SMD**
Age group						
20 - 24	<0.2	7.1	-0.37	3.3	8.4	-0.22
25 - 29	0.3	7.2	-0.37	5.7	8.5	-0.11
30 - 34	0.4	5.9	-0.32	21.2	7.0	0.41
35 - 39	0.7	6.3	-0.31	5.2	7.4	-0.09
40 - 44	1.1	6.8	-0.30	7.7	7.9	-0.01
45 - 49	0.9	7.0	-0.32	3.8	8.0	-0.18
50 - 54	1.6	8.0	-0.30	10.0	8.8	0.04
55 - 59	3.3	7.7	-0.19	10.0	8.2	0.06
60 - 64	7.3	9.3	-0.07	5.2	9.3	-0.16
65 - 69	9.1	7.8	0.05	5.2	7.3	-0.08
75 - 79	15.3	4.9	0.35	4.2	3.4	0.04
80 - 84	19.8	6.1	0.42	4.7	3.9	0.04
85 - 89	17.5	5.5	0.38	3.7	3.5	0.01
90 - 94	7.9	3.2	0.21	3.2	2.2	0.06
95 - 99	1.8	1.0	0.07	1.4	0.7	0.07
100 - 104	0.3	0.2	0.03	0.3	0.1	0.04
Gender: Female	49.7	52.2	-0.05	55.2	52.0	0.06
Medical history: General						
Acute respiratory disease	53.9	49.8	0.08	69.1	50.9	0.37
Chronic liver disease	8.2	4.0	0.17	4.3	3.6	0.03
Chronic obstructive lung disease	10.1	2.7	0.31	2.3	1.8	0.03

Crohn's disease	0.3	0.1	0.04	<0.2	0.1	-0.03
Dementia	45.1	17.1	0.64	12.4	10.5	0.06
Depressive disorder	33.4	16.0	0.41	17.6	12.3	0.15
Diabetes mellitus	46.8	21.8	0.55	17.2	18.0	-0.02
Gastroesophageal reflux disease	50.8	30.1	0.43	33.8	28.6	0.11
Gastrointestinal hemorrhage	8.0	2.8	0.23	5.7	2.2	0.18
Hyperlipidemia	70.3	34.3	0.77	38.4	29.7	0.18
Hypertensive disorder	84.3	37.1	1.10	37.7	29.5	0.18
Lesion of liver	5.7	2.9	0.14	4.8	2.7	0.11
Obesity	0.2	0.2	0.01	0.5	0.2	0.06
Osteoarthritis	24.1	14.5	0.25	11.2	13.7	-0.08
Pneumonia	27.6	11.6	0.41	8.5	9.1	-0.02
Psoriasis	0.8	0.8	0.01	0.6	0.7	-0.02
Renal impairment	21.8	4.6	0.52	5.9	3.0	0.14
Rheumatoid arthritis	2.8	1.5	0.09	2.5	1.4	0.08
Schizophrenia	2.5	3.6	-0.06	1.9	3.6	-0.10
Ulcerative colitis	0.5	0.2	0.06	0.7	0.1	0.09
Urinary tract infectious disease	19.7	7.7	0.36	7.9	5.9	0.08
Visual system disorder	41.4	31.7	0.20	53.6	31.3	0.46
Medical history: Cardiovascular disease						
Cerebrovascular disease	17.9	5.7	0.38	2.3	2.2	0.00
Peripheral vascular disease	20.2	9.7	0.30	8.6	7.9	0.02
Pulmonary embolism	7.1	1.2	0.30	1.8	0.9	0.08
Venous thrombosis	7.8	2.8	0.23	6.1	2.4	0.18
Medical history: Neoplasms						
Hematologic neoplasm	1.4	0.5	0.09	0.4	0.5	-0.01

Malignant lymphoma	0.5	0.2	0.06	<0.2	0.2	-0.01
Malignant neoplasm of anorectum	0.8	0.3	0.07	0.3	0.3	0.00
Malignant neoplastic disease	12.5	5.9	0.23	10.4	5.3	0.19
Malignant tumor of breast	0.3	0.6	-0.05	<0.2	0.7	-0.10
Malignant tumor of colon	1.2	0.5	0.08	0.4	0.4	0.00
Malignant tumor of lung	0.8	0.4	0.06	<0.2	0.3	-0.02
Malignant tumor of urinary bladder	0.4	0.2	0.04	2.6	0.2	0.21
Primary malignant neoplasm of prostate	2.2	0.6	0.14	0.5	0.5	0.01

Data are presented as %.

PS, propensity score; AF, atrial fibrillation; SMD, standardized mean difference

If the absolute value of SMD is less than 0.1 after PS adjustment, it is considered that baseline difference is adequately balanced

Table S3. The baseline characteristics of patients analyzed for acute myocardial infarction within 30 days of a COVID-19 diagnosis.

	Before PS adjustment			After PS adjustment		
	History of AF (N=1,959)	No history of AF (N=106,387)	SMD	History of AF (N=1,959)	No history of AF (N=106,387)	SMD
Age group						
20 - 24	<0.2	7.1	-0.37	0.8	7.1	-0.32
25 - 29	0.3	7.2	-0.37	15.8	7.2	0.27
30 - 34	0.4	5.9	-0.32	11.0	6.0	0.18
35 - 39	0.7	6.3	-0.31	1.8	6.3	-0.23
40 - 44	1.1	6.8	-0.30	6.1	6.8	-0.03
45 - 49	0.9	7.0	-0.32	5.4	7.0	-0.07
50 - 54	1.6	8.0	-0.30	9.0	8.0	0.04
55 - 59	3.3	7.7	-0.19	8.0	7.7	0.01
60 - 64	7.3	9.3	-0.07	7.2	9.2	-0.07
65 - 69	9.1	7.8	0.05	5.6	7.8	-0.09
70 - 74	12.5	6.1	0.22	6.0	6.2	-0.01
75 - 79	15.3	4.9	0.35	5.3	4.9	0.02
80 - 84	19.8	6.1	0.42	6.8	6.1	0.03
85 - 89	17.5	5.5	0.38	5.6	5.5	0.01
90 - 94	7.9	3.2	0.21	4.3	3.2	0.06
95 - 99	1.8	1.0	0.07	1.1	1.0	0.01
100 - 104	0.3	0.2	0.03	0.2	0.2	0.00
Gender: Female	49.7	52.2	-0.05	43.0	52.4	-0.19
Medical history: General						
Acute respiratory disease	53.9	49.8	0.08	54.2	49.8	0.09
Chronic liver disease	8.2	4.0	0.17	4.0	4.0	0.00

Chronic obstructive lung disease	10.1	2.7	0.31	3.3	2.7	0.04
Crohn's disease	0.3	0.1	0.04	<0.1	0.1	-0.01
Dementia	45.1	17.1	0.64	19.1	17.1	0.05
Depressive disorder	33.4	16.0	0.41	16.5	15.9	0.01
Diabetes mellitus	46.8	21.8	0.55	19.8	21.7	-0.04
Gastroesophageal reflux disease	50.8	30.1	0.43	26.4	30.1	-0.08
Gastrointestinal hemorrhage	8.0	2.8	0.23	3.4	2.7	0.04
Hyperlipidemia	70.3	34.3	0.77	33.5	34.2	-0.01
Hypertensive disorder	84.3	37.1	1.10	39.2	37.1	0.04
Lesion of liver	5.7	2.9	0.14	3.9	2.9	0.06
Obesity	0.2	0.2	0.01	0.6	0.2	0.06
Osteoarthritis	24.1	14.5	0.25	11.7	14.5	-0.08
Pneumonia	27.6	11.6	0.41	11.2	11.5	-0.01
Psoriasis	0.8	0.8	0.01	0.5	0.8	-0.04
Renal impairment	21.8	4.6	0.52	7.0	4.5	0.11
Rheumatoid arthritis	2.8	1.5	0.09	1.5	1.5	0.00
Schizophrenia	2.5	3.6	-0.06	2.2	3.6	-0.09
Ulcerative colitis	0.5	0.2	0.06	0.6	0.2	0.07
Urinary tract infectious disease	19.7	7.7	0.36	10.7	7.6	0.11
Visual system disorder	41.4	31.7	0.20	43.1	31.7	0.24
Medical history: Cardiovascular disease						
Cerebrovascular disease	17.9	5.7	0.38	6.7	5.7	0.04
Peripheral vascular disease	20.2	9.7	0.30	11.6	9.6	0.06
Pulmonary embolism	7.1	1.2	0.30	2.0	1.2	0.06
Venous thrombosis	7.8	2.8	0.23	3.4	2.8	0.04
Medical history: Neoplasms						

Hematologic neoplasm	1.4	0.5	0.09	0.7	0.5	0.02
Malignant lymphoma	0.5	0.2	0.06	0.2	0.2	0.01
Malignant neoplasm of anorectum	0.8	0.3	0.07	0.2	0.3	-0.02
Malignant neoplastic disease	12.5	5.9	0.23	8.9	5.9	0.11
Malignant tumor of breast	0.3	0.6	-0.05	<0.1	0.7	-0.09
Malignant tumor of colon	1.2	0.5	0.08	0.5	0.5	0.00
Malignant tumor of lung	0.8	0.4	0.06	0.3	0.4	-0.02
Malignant tumor of urinary bladder	0.4	0.2	0.04	0.3	0.2	0.02
Primary malignant neoplasm of prostate	2.2	0.6	0.14	0.8	0.6	0.03

Data are presented as %.

PS, propensity score; AF, atrial fibrillation; SMD, standardized mean difference

If the absolute value of SMD is less than 0.1 after PS adjustment, it is considered that baseline difference is adequately balanced

Table S4. The baseline characteristics of patients analyzed for acute myocardial infarction at more than 30 days after a COVID-19 diagnosis.

	Before PS adjustment			After PS adjustment		
	**History of AF (N=1,747)**	**No history of AF (N=103,126)**	**SMD**	**History of AF (N=1,747)**	**No history of AF (N=103,126)**	**SMD**
Age group						
20 - 24	<0.2	7.1	-0.37	2.3	7.3	-0.24
25 - 29	0.3	7.2	-0.37	13.0	7.4	0.18
30 - 34	0.4	5.9	-0.32	11.2	6.1	0.18
35 - 39	0.7	6.3	-0.31	4.3	6.5	-0.10
40 - 44	1.1	6.8	-0.30	7.3	7.0	0.01
45 - 49	0.9	7.0	-0.32	2.9	7.2	-0.20
50 - 54	1.6	8.0	-0.30	11.0	8.2	0.10
55 - 59	3.3	7.7	-0.19	8.2	7.8	0.01
60 - 64	7.3	9.3	-0.07	5.8	9.4	-0.14
65 - 69	9.1	7.8	0.05	5.9	7.8	-0.08
70 - 74	12.5	6.1	0.22	6.1	6.1	0.00
75 - 79	15.3	4.9	0.35	4.8	4.7	0.00
80 - 84	19.8	6.1	0.42	6.6	5.8	0.04
85 - 89	17.5	5.5	0.38	5.1	5.0	0.00
90 - 94	7.9	3.2	0.21	3.8	2.8	0.06
95 - 99	1.8	1.0	0.07	1.5	0.8	0.06
100 - 104	0.3	0.2	0.03	0.2	0.1	0.02
Gender: Female	49.7	52.2	-0.05	44.4	52.3	-0.16
Medical history: General						
Acute respiratory disease	53.9	49.8	0.08	61.6	49.8	0.24
Chronic liver disease	8.2	4.0	0.17	4.5	3.9	0.03

Chronic obstructive lung disease	10.1	2.7	0.31	3.2	2.4	0.05
Crohn's disease	0.3	0.1	0.04	<0.1	0.1	-0.01
Dementia	45.1	17.1	0.64	17.7	15.8	0.05
Depressive disorder	33.4	16.0	0.41	20.6	15.4	0.14
Diabetes mellitus	46.8	21.8	0.55	21.1	20.9	0.00
Gastroesophageal reflux disease	50.8	30.1	0.43	30.9	29.8	0.02
Gastrointestinal hemorrhage	8.0	2.8	0.23	5.6	2.6	0.15
Hyperlipidemia	70.3	34.3	0.77	42.4	33.6	0.18
Hypertensive disorder	84.3	37.1	1.10	42.7	35.9	0.14
Lesion of liver	5.7	2.9	0.14	4.1	2.8	0.07
Obesity	0.2	0.2	0.01	0.4	0.2	0.05
Osteoarthritis	24.1	14.5	0.25	13.5	14.4	-0.03
Pneumonia	27.6	11.6	0.41	10.2	10.6	-0.01
Psoriasis	0.8	0.8	0.01	1.6	0.8	0.08
Renal impairment	21.8	4.6	0.52	6.7	4.0	0.12
Rheumatoid arthritis	2.8	1.5	0.09	2.9	1.5	0.09
Schizophrenia	2.5	3.6	-0.06	2.1	3.6	-0.09
Ulcerative colitis	0.5	0.2	0.06	1.8	0.1	0.17
Urinary tract infectious disease	19.7	7.7	0.36	9.1	7.2	0.07
Visual system disorder	41.4	31.7	0.20	43.1	31.7	0.24
Medical history: Cardiovascular disease						
Cerebrovascular disease	17.9	5.7	0.38	7.1	5.4	0.07
Peripheral vascular disease	20.2	9.7	0.30	9.1	9.4	-0.01
Pulmonary embolism	7.1	1.2	0.30	2.0	1.1	0.07
Venous thrombosis	7.8	2.8	0.23	5.6	2.7	0.15
Medical history: Neoplasms						

Hematologic neoplasm	1.4	0.5	0.09	0.5	0.5	0.00
Malignant lymphoma	0.5	0.2	0.06	0.1	0.2	-0.01
Malignant neoplasm of anorectum	0.8	0.3	0.07	0.2	0.3	-0.01
Malignant neoplastic disease	12.5	5.9	0.23	8.2	5.6	0.10
Malignant tumor of breast	0.3	0.6	-0.05	<0.1	0.7	-0.09
Malignant tumor of colon	1.2	0.5	0.08	0.5	0.4	0.00
Malignant tumor of lung	0.8	0.4	0.06	0.2	0.3	-0.02
Malignant tumor of urinary bladder	0.4	0.2	0.04	1.5	0.2	0.14
Primary malignant neoplasm of prostate	2.2	0.6	0.14	0.8	0.5	0.03

Data are presented as %.

PS, propensity score; AF, atrial fibrillation; SMD, standardized mean difference

If the absolute value of SMD is less than 0.1 after PS adjustment, it is considered that baseline difference is adequately balanced

Table S5. The baseline characteristics of patients analyzed for stroke within 30 days of a COVID-19 diagnosis.

	Before PS adjustment			After PS adjustment		
	History of AF (N=1,109)	No history of AF (N=93,722)	SMD	History of AF (N=1,109)	No history of AF (N=93,722)	SMD
Age group						
20 - 24	<0.2	7.1	-0.37	0.8	8.0	-0.36
25 - 29	0.3	7.2	-0.37	10.1	8.2	0.07
30 - 34	0.4	5.9	-0.32	21.2	6.8	0.42
35 - 39	0.7	6.3	-0.31	3.6	7.1	-0.16
40 - 44	1.1	6.8	-0.30	5.0	7.6	-0.11
45 - 49	0.9	7.0	-0.32	5.5	7.8	-0.09
50 - 54	1.6	8.0	-0.30	8.1	8.6	-0.02
55 - 59	3.3	7.7	-0.19	10.2	8.1	0.07
60 - 64	7.3	9.3	-0.07	7.6	9.3	-0.06
65 - 69	9.1	7.8	0.05	4.8	7.4	-0.11
70 - 74	12.5	6.1	0.22	5.2	5.4	-0.01
75 - 79	15.3	4.9	0.35	3.8	3.7	0.00
80 - 84	19.8	6.1	0.42	5.2	4.3	0.04
85 - 89	17.5	5.5	0.38	4.4	4.0	0.02
90 - 94	7.9	3.2	0.21	3.2	2.6	0.04
95 - 99	1.8	1.0	0.07	0.9	0.9	0.01
Gender: Female	49.7	52.2	-0.05	50.9	52.0	-0.02
Medical history: General						
Acute respiratory disease	53.9	49.8	0.08	61.2	51.0	0.20
Chronic liver disease	8.2	4.0	0.17	4.0	3.8	0.01
Chronic obstructive lung disease	10.1	2.7	0.31	2.7	2.1	0.04

Crohn's disease	0.3	0.1	0.04	<0.2	0.1	-0.03
Dementia	45.1	17.1	0.64	12.7	11.8	0.03
Depressive disorder	33.4	16.0	0.41	13.7	13.0	0.02
Diabetes mellitus	46.8	21.8	0.55	18.1	19.1	-0.03
Gastroesophageal reflux disease	50.8	30.1	0.43	24.9	29.2	-0.10
Gastrointestinal hemorrhage	8.0	2.8	0.23	3.5	2.3	0.07
Hyperlipidemia	70.3	34.3	0.77	32.3	31.0	0.03
Hypertensive disorder	84.3	37.1	1.10	32.8	31.4	0.03
Lesion of liver	5.7	2.9	0.14	3.9	2.8	0.06
Obesity	0.2	0.2	0.01	0.4	0.2	0.04
Osteoarthritis	24.1	14.5	0.25	10.6	13.9	-0.10
Pneumonia	27.6	11.6	0.41	8.7	10.0	-0.04
Psoriasis	0.8	0.8	0.01	0.6	0.7	-0.01
Renal impairment	21.8	4.6	0.52	7.3	3.6	0.16
Rheumatoid arthritis	2.8	1.5	0.09	1.7	1.4	0.02
Schizophrenia	2.5	3.6	-0.06	2.0	3.6	-0.10
Ulcerative colitis	0.5	0.2	0.06	0.7	0.1	0.09
Urinary tract infectious disease	19.7	7.7	0.36	5.9	6.3	-0.01
Visual system disorder	41.4	31.7	0.20	51.2	31.5	0.40
Medical history: Cardiovascular disease						
Cerebrovascular disease	17.9	5.7	0.38	2.4	2.5	0.00
Peripheral vascular disease	20.2	9.7	0.30	7.6	8.4	-0.03
Pulmonary embolism	7.1	1.2	0.30	2.3	1.0	0.10
Venous thrombosis	7.8	2.8	0.23	4.2	2.6	0.09
Medical history: Neoplasms						
Hematologic neoplasm	1.4	0.5	0.09	0.5	0.5	0.00

Malignant lymphoma	0.5	0.2	0.06	<0.2	0.2	-0.02
Malignant neoplastic disease	12.5	5.9	0.23	9.2	5.6	0.14
Malignant tumor of breast	0.3	0.6	-0.05	<0.2	0.7	-0.10
Malignant tumor of colon	1.2	0.5	0.08	0.3	0.4	-0.01
Malignant tumor of lung	0.8	0.4	0.06	0.3	0.3	0.00
Malignant tumor of urinary bladder	0.4	0.2	0.04	0.6	0.2	0.07
Primary malignant neoplasm of prostate	2.2	0.6	0.14	0.6	0.5	0.01

Data are presented as %.

PS, propensity score; AF, atrial fibrillation; SMD, standardized mean difference

If the absolute value of SMD is less than 0.1 after PS adjustment, it is considered that baseline difference is adequately balanced

Table S6. The baseline characteristics of patients analyzed for stroke at more than 30 days after a COVID-19 diagnosis.

	**Before PS adjustment**			**After PS adjustment**		
	**History of AF (N=995)**	**No history of AF (N=91,501)**	**SMD**	**History of AF (N=995)**	**No history of AF (N=91,501)**	**SMD**
Age group						
20 - 24	<0.2	7.1	-0.37	3.3	8.2	-0.22
25 - 29	0.3	7.2	-0.37	13.1	8.4	0.15
30 - 34	0.4	5.9	-0.32	11.3	6.9	0.15
35 - 39	0.7	6.3	-0.31	5.6	7.3	-0.07
40 - 44	1.1	6.8	-0.30	7.4	7.8	-0.02
45 - 49	0.9	7.0	-0.32	3.7	8.0	-0.18
50 - 54	1.6	8.0	-0.30	11.7	8.8	0.10
55 - 59	3.3	7.7	-0.19	9.7	8.2	0.06
60 - 64	7.3	9.3	-0.07	5.3	9.4	-0.16
65 - 69	9.1	7.8	0.05	5.2	7.4	-0.09
70 - 74	12.5	6.1	0.22	5.5	5.3	0.01
75 - 79	15.3	4.9	0.35	4.3	3.6	0.04
80 - 84	19.8	6.1	0.42	4.8	4.1	0.04
85 - 89	17.5	5.5	0.38	4.2	3.6	0.03
90 - 94	7.9	3.2	0.21	3.0	2.3	0.05
95 - 99	1.8	1.0	0.07	1.5	0.7	0.07
100 - 104	0.3	0.2	0.03	0.3	0.1	0.04
Gender: Female	49.7	52.2	-0.05	44.4	51.9	-0.15
Medical history: General						
Acute respiratory disease	53.9	49.8	0.08	69.2	51.1	0.38
Chronic liver disease	8.2	4.0	0.17	4.6	3.7	0.05

Chronic obstructive lung disease	10.1	2.7	0.31	2.4	1.9	0.03
Crohn's disease	0.3	0.1	0.04	<0.2	0.1	-0.03
Dementia	45.1	17.1	0.64	12.9	10.9	0.06
Depressive disorder	33.4	16.0	0.41	17.6	12.5	0.14
Diabetes mellitus	46.8	21.8	0.55	18.4	18.6	0.00
Gastroesophageal reflux disease	50.8	30.1	0.43	36.3	29.0	0.16
Gastrointestinal hemorrhage	8.0	2.8	0.23	5.8	2.2	0.18
Hyperlipidemia	70.3	34.3	0.77	41.4	30.5	0.23
Hypertensive disorder	84.3	37.1	1.10	38.7	30.3	0.17
Lesion of liver	5.7	2.9	0.14	4.7	2.7	0.11
Obesity	0.2	0.2	0.01	0.5	0.2	0.05
Osteoarthritis	24.1	14.5	0.25	11.8	13.8	-0.06
Pneumonia	27.6	11.6	0.41	8.8	9.4	-0.02
Psoriasis	0.8	0.8	0.01	0.6	0.7	-0.02
Renal impairment	21.8	4.6	0.52	6.5	3.2	0.15
Rheumatoid arthritis	2.8	1.5	0.09	2.4	1.4	0.07
Schizophrenia	2.5	3.6	-0.06	1.9	3.6	-0.10
Ulcerative colitis	0.5	0.2	0.06	0.7	0.1	0.09
Urinary tract infectious disease	19.7	7.7	0.36	8.2	6.0	0.09
Visual system disorder	41.4	31.7	0.20	43.3	31.5	0.24
Medical history: Cardiovascular disease						
Cerebrovascular disease	17.9	5.7	0.38	2.9	2.3	0.04
Peripheral vascular disease	20.2	9.7	0.30	9.0	8.2	0.03
Pulmonary embolism	7.1	1.2	0.30	2.1	0.9	0.09
Venous thrombosis	7.8	2.8	0.23	6.0	2.5	0.17
Medical history: Neoplasms						

Hematologic neoplasm	1.4	0.5	0.09	0.4	0.5	-0.01
Malignant lymphoma	0.5	0.2	0.06	<0.2	0.2	-0.02
Malignant neoplasm of anorectum	0.8	0.3	0.07	0.3	0.3	0.00
Malignant neoplastic disease	12.5	5.9	0.23	10.5	5.4	0.19
Malignant tumor of breast	0.3	0.6	-0.05	<0.2	0.7	-0.10
Malignant tumor of colon	1.2	0.5	0.08	0.4	0.4	-0.01
Malignant tumor of urinary bladder	0.4	0.2	0.04	2.6	0.2	0.21
Primary malignant neoplasm of prostate	2.2	0.6	0.14	0.5	0.5	0.01

Data are presented as %.

PS, propensity score; AF, atrial fibrillation; SMD, standardized mean difference

If the absolute value of SMD is less than 0.1 after PS adjustment, it is considered that baseline difference is adequately balanced
