# Supplementary figures and images for: Long-Term Clinical Implications of Atrial Fibrillation on Mortality in Patients Hospitalized with COVID-19: A Nationwide Cohort Study
Source: J Clin Med. 2023 Oct 13;12(20):6504. doi: 10.3390/jcm12206504 (PMC10607130; doi:10.3390/jcm12206504)

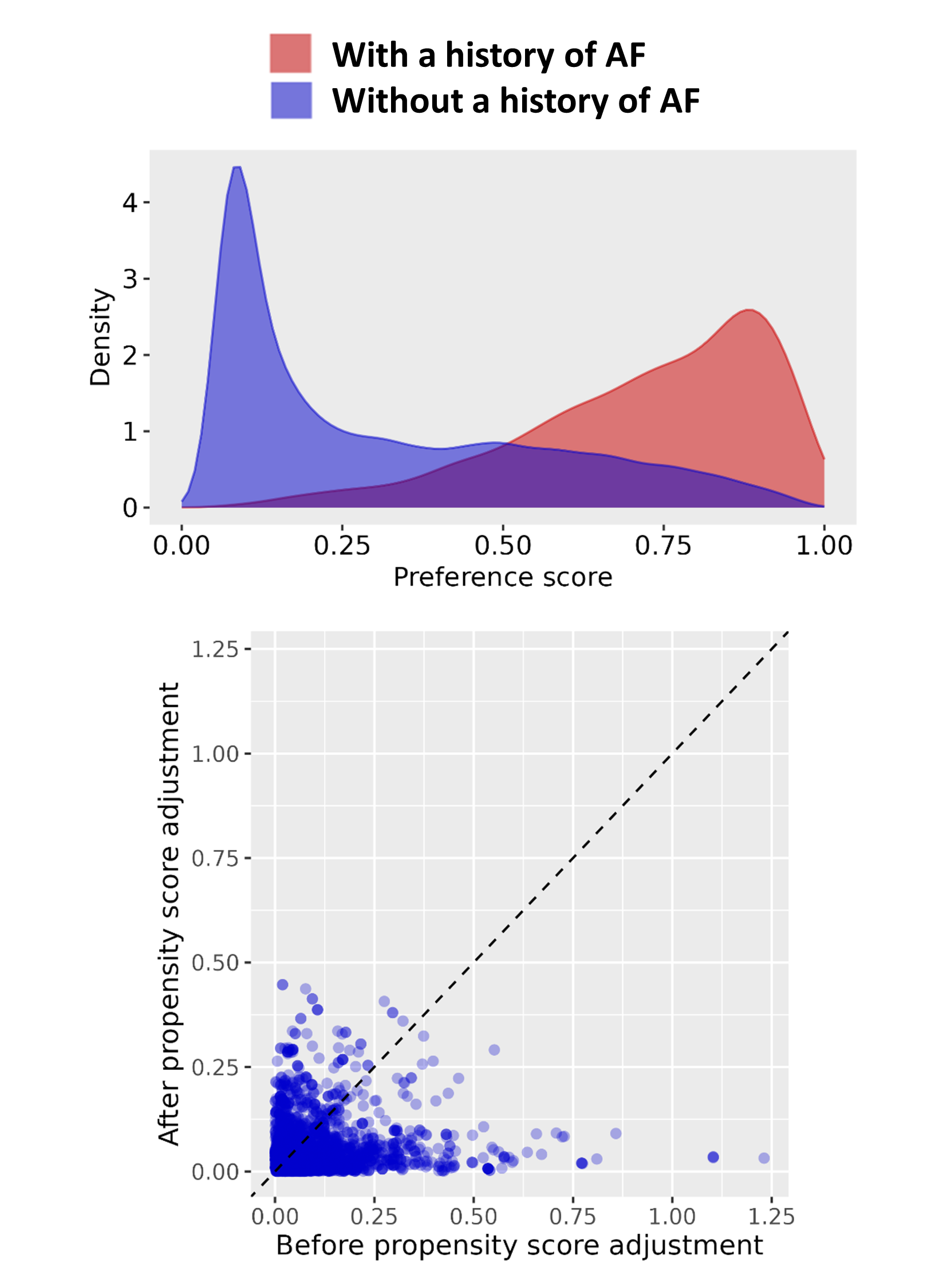

Supplement: Supplementary file 1 [file jcm-12-06504-s001.zip › Suppl_FigureS1_AF_COVID.tif]

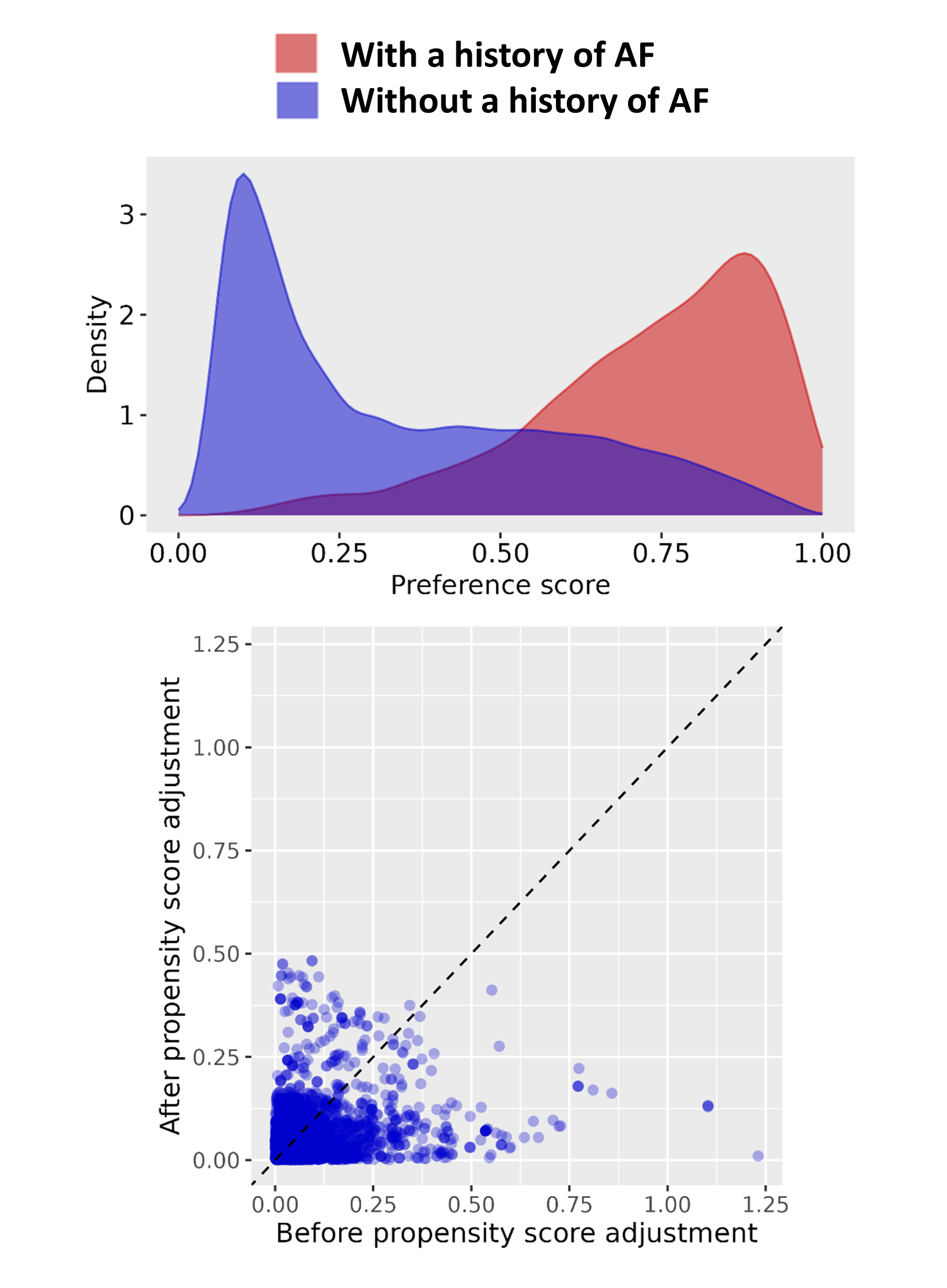

Supplement: Supplementary file 1 [file jcm-12-06504-s001.zip › Suppl_FigureS2_AF_COVID.tif]
